# Thirty-day mortality after hip fractures: has anything changed?

**DOI:** 10.1007/s00590-016-1744-4

**Published:** 2016-03-04

**Authors:** Dionysios Giannoulis, Giorgio M. Calori, Peter V. Giannoudis

**Affiliations:** Academic Department of Trauma and Orthopaedics Surgery, School of Medicine, University of Leeds, Leeds, UK; Academic Department of Trauma and Orthopaedics, School of Medicine, University of Milan, Milan, Italy; NIHR Leeds Biomedical Research Unit, Chapel Allerton Hospital, Leeds, West Yorkshire, LS7 4SA UK

**Keywords:** Hip fracture, Complications, Mortality, Early surgery, Fast-track pathways

## Abstract

Bone density insufficiency is the main cause for significant musculoskeletal trauma in the elderly population following low-energy falls. Hip fractures, in particular, represent an important public health concern taking into account the complicated needs of the patients due to their medical comorbidities as well as their rehabilitation and social demands. The annual cost for the care of these patients is estimated at around 2 billion pounds (£) in the UK and is ever growing. An increased early and late mortality rate is also recognised in these injuries together with significant adversities for the patients. Lately, in order to improve the outcomes of this special cohort of patients, fast-track care pathways and government initiatives have been implemented. It appears that these measures have contributed in a steady year-by-year reduction of the 30-day mortality rates. Whether we have currently reached a plateau or whether an ongoing reduction in mortality rates will continue to be observed is yet to be seen.

## Introduction

Hip fractures continue to be a significant injury in the elderly population [1]. They represent an important public health concern taking into account the complicated needs of the patients due to their medical comorbidities as well as their rehabilitation and social demands [2].

Data from studies that report a rise in the number of the elderly in the future predict also an increase in the number of hip injuries that will require treatment [3]. In the USA, elderly are the fastest growing category of the population with people above the age of 65 years or age expected to reach an estimated value of 89 million by year 2050 [4]. In the UK, by 2020 there is an estimate of 101,000 annual cases of patients with hip fractures [5]. The majority of the patients with hip fractures are women (approximately 75 %) with an average age of 80 years [6].

Hip fractures are associated with increased morbidity and significant financial cost implications [7]. The annual cost for the care of these patients in the UK is estimated at around 2 billion pounds (£) and is ever growing [8]. In the USA, approximately $40,000 are spent in the first year for every patient with hip fracture for direct medical costs [9]. An increased early and late mortality rate is also recognised in these injuries together with significant adversities for these patients. Therefore, a well-organised multidirectional approach to this hazardous medical entity is essential in order to improve the outcomes in this cohort of elderly patients [10].

Despite the fact that significant evolution has been achieved in the surgical approaches and patient care lately, there is obscure evidence whether mortality rates are improving [9]. The results in the literature from studies generated from the existing hip fracture registries are conflicting. For instance, in a study by Daugaard et al. from 2003 to 2010, the 30-day mortality rate was reported to be 10 % in 38,020 patients, whereas in another study by Caretta et al. from 2004 to 2007, the 30-day mortality rate was 3.5 % in 1320 patients [11, 12]. With the implementation of specific hip fracture pathways to enhance the care of these frail elderly patients, one would argue that improvements in the morbidity and mortality rates should prevail.

The aim of this study therefore is to investigate whether the overall initiatives introduced during the past few years to improve the care of elderly patients with hip fractures have made an impact on the early (30 day) mortality rate.

## Patients and methods

A literature review of relevant publications was carried out for the past 15 years. We used such ‘mesh words’ as early mortality rate, hip fractures, elderly, delay to surgery, 30-day mortality, early complications. Inclusion criteria were articles in English language, original papers published in peer-review journals that contained data about 30-day mortality after traumatic hip fractures in the elderly. Exclusion criteria were case reports, editorials, review articles and articles of hip fractures in young adults or children, articles about stress fractures of the proximal femur, as well as articles in other languages than in English. Moreover, we analysed the data of our hospital hip fracture database of elderly patients treated in our tertiary referral centre during the past 3 years.

It is of note that multiple variables are described in the literature to play an indispensable role in the 30-day mortality rates in different studies, although these variables are not encountered in every single study. Two major categories can be identified in these variables: variables that cannot be altered and referred to fixed patient characteristics and variables that can be changed and controlled [13]. Age, fracture type, gender, pre-fracture condition and mobility, date of admission (weekend or week days), mechanism of injury, accommodation and the Society of Anaesthesiologists score (ASA) are some of the parameters that cannot be altered, whereas grade of responsible surgeon and anaesthetist, time from the injury to operation, time from admission to operation and type of anaesthesia belong to the changing variables [11, 14, 15].

## Results

Initially 54 manuscripts were found to be relevant to this subject. However, following the application of the inclusion and exclusion criteria 15 manuscripts [9, 11–13, 16–26] and data from 11 annual reports of the National Hip Fracture Database (NHFD) formed the basis of this review.

From the data obtained, it appears that conflicting reports are recognised in many cases. For instance, Choi et al. found a very low (1.4 %) 30-day mortality rate, whereas Daugaard et al. noted an early mortality rate of 10 % [11, 21]. Nonetheless, the majority of the most recent studies report that lately with the implementation of new guidelines and treatment protocols, a trend of a continuous decrease in the 30-day mortality rate of patients with hip fractures has been observed (Table 1). In agreement with this observation are the findings from the UK (based on the NHFD) (the largest hip fracture database in the world) [6]. A continuous steady reduction in the 30-day mortality rate has been shown as a result of the new multidisciplinary approach provided to the patients, the fastest time to surgery, the input in the medical treatment of the patients by the orthogeriatricians and the implementation of NICE guidelines for hip fractures (Table 2). Data obtained from our institution with regard to the 30-day mortality rate after hip fractures also demonstrated this trend (Table 3).

**Table 1:** Data collected from international studies providing information about 30-day mortality rate after hip fracture

Authors	Year	No of patients	Early mortality rate (%)
Charletton et al.	2006–2013	4426	6.5–12.1
Inacio et al.	2009–2011	12,562	6.2
Tarrant et al.	2011	437	8.4
Khan et al.	2009–2010	467	7.5
Lau et al.	2009–2010	759	2.5
Daugaard et al.	2003–2010	38,020	10
Choi et al.	2002–2009	874	1.4
Miller et al.	2006–2008	338,092	8.1
Castronuovo et al.	2006–2007	6896	7
Caretta et al.	2004–2007	1320	3.5
Nielsen et al.	2005–2006	6266	10.3
Brauer et al.	1986–2005	786,717	Female: 5.9–5.2 Male: 11.9–9.3
Holt et al.	1998–2004	18,817	7
Moran et al.	1999–2003	2660	9
Gini et al. (Lazio)	1999–2003	32,019	5
Gini et al. (Tuscany)	1999–2003	30,406	2.8

**Table 2:** Data collected from the National Hip Fracture Database (NHFD) in regard to 30-day mortality after hip fracture

Author	Year	No of patients	30-day mortality rate (%)
NHFD	2013	64,838	8.02
NHFD	2012	61,508	8.2
NHFD	2011	53,879	8.5
NHFD	2010	54,129	8.8
NHFD	2009	53,427	9.2
NHFD	2008	52,600	10.4
NHFD	2007	52,435	10.9
NHFD	2006	50,732	11.3
NHFD	2005	51,408	11.4
NHFD	2004	50,995	11.0
NHFD	2003	51,985	11.5

As noted there is a continuous decrease in the 30-day mortality rate

**Table 3:** Data regarding the 30-day mortality rate of patients treated in the Leeds General Infirmary (LGI)

LGI	Year	Hip admissions	Deaths at 30 days	30-day mortality rate (%)
NHFD	2015 (till November)	638	44	6.7
NHFD	2014	715	50	6.9
NHFD	2013	678	74	10

## Discussion

Patients suffering from a hip fracture have a significantly higher early- and long-term mortality rate after the initial injury compared with healthy individuals of the same age with no fracture [19]. Currently, no consensus has been reached with regard to the factors responsible for this difference in the mortality rate. This lack of consensus exists also in the established protocols for optimisation of patient care, the definition of early and late surgery, the implementation of rehabilitation protocols, the length of hospital stay, thus making it extremely difficult to compare data from registries with different diagnostic and treatment pathways. In a study of 18,817 patients by Holt et al. [13] in the Scottish Hip Fracture Audit Database, male sex, increased age and preoperative comorbidities (according to ASA) were all connected with a higher death risk. In the same study variables that can be changed in a patient suffering a hip fracture such as time to theatre and grade of surgeon and anaesthesiologist did not seem to affect the mortality rate in a significant way, whereas the period from the injury to the operation (and not the time from the hospital admission to the surgery) had a significant effect on 30-day mortality [13]. In an additional study from Holt et al. [27] 2 years later including 4284 patients the conclusion that patients with severe medical comorbidities were more likely to be operated late and faced an increased 30-day mortality rate was reported.

Noteworthy, White et al. [3] highlighted that despite medical advancements and the implementation of new fast-track protocols, a rise in the 30-day mortality rate after surgical treatment of hip fractures should be expected from 8.3 to 8.9–9.3 %, adding 2200–7000 deaths every year in the near future in the UK. The authors justified their projection on the following parameters: increased age of the population in the near future and increased age-related comorbidities [3].

In a recent pilot study from the Hip Fracture Accelerated Surgical Treatment and Care Track (HIP ATTACK) investigators, patients who have been treated in the first 6 h after the injury (accelerated care group) had significantly lower 30-day mortality rate compared to the standard care group (3 and 13 %, respectively) (the mean time to surgery for the accelerated group was 6 h compared to the 24, 2 h for the standard group) [28]. This was attributed to a very aggressive early medical optimisation and early operative treatment of the hip fracture [28].

With regard to the impact on mortality of the date of admission parameter, reports from the literature are contradictive. In a study by Clarke et al. in 2010, no significant difference was found between admission on weekdays and weekends, whereas Schilling et al. in 2010 reported an increased mortality rate for weekend admissions in a total of 166,920 patients 20 % of whom suffered from hip fractures [29, 30].

Guidelines established in New Zealand and Scottish Health Care System recommend early surgery (within the first 24 h of the injury) for the reduction of postoperative complications and mortality [31, 32]. However, the reports in the literature seem to be controversial. On the one hand, there is a meta-analysis performed by Shiga et al. that reported a 41 % increase in the 30-day mortality rate and a 32 % increase in the 1-year mortality rate after delayed surgery for a patient with a hip fracture, but on the other hand, there are several studies that did not demonstrate a connection between the treatment time and postoperative complications and mortality [26, 33, 34]. With regard to the development of complications, the respiratory system, the cardiovascular system, the urogenital and the central nervous system are mostly involved, with the operation to have a significant stress to all of the systems [19].

In a systematic review of 52 published studies that included 291,413 patients in total, a suggestion was made that hip surgery should take place within the first 48 h after injury in order to expect a reduction in the rate of complications and overall mortality [19]. The risk of 30-day mortality in a Danish study of 38,020 patients with hip fracture increased with an OR of 1.3 per 24-h surgical delay [11]. Similarly, in a recent study by Nyholm et al. [35] even a delay in the operation for more than 12 h increased the 30-day mortality rate compared to the patients operated within 12 h from the injury. It is of interest that another study from Sweden showed that longer length of hospital stay seems to be beneficial for patients with regard to early mortality rates [36].

For the type of anaesthesia and the possible connection to early mortality rate, a meta-analysis of 22 trials performed by Parker et al. [37] including 2567 patients could not report a safe conclusion due to insufficient evidence available from the studies.

Considerable debate has been noted also in the grade of the orthopaedic surgeon and the anaesthetist treating patients with hip fractures. There have been data suggesting that patients treated by junior anaesthetists experience lower 30-day mortality rates than other patients treated by senior staff [13, 27]. However, this finding could be connected with the fact that senior staff is more likely to handle patients with significant comorbidities [13]. In the same study, when the results in patient care mix were calculated with the multivariate model, no significant difference was noted in the 30-day mortality regarding the staff grade [13].

It is of interest that there is no clear consensus in the literature with regard to the early surgery definition. A hospital statistics analysis of 129,522 patients performed by Bottle et al. [38] showed a higher risk of 30-day mortality in patients operated after 24 h. In another study by McGuire et al. [39] in 18,209 patients with hip fracture increased early mortality rate was reported for patients receiving delayed surgery (after 48 h from the initial injury). On the other hand, there is evidence to support that early surgery does not play an important role in short-term mortality rates. For instance, Holt et al. [13, 27] reported that no clear effect of the time of operation and 30-day mortality could be found, with the time to surgery to be representing a chance association. Therefore, new studies are essential in order to be able to specify whether the over 48-h operation delay is an independent risk factor for increased mortality and also the relationship between time to operation and early mortality in the case mix [40].

Preoperative factors that can influence postoperative complications and mortality such as increased age, poor pre-injury functional level, multiple medical comorbidities and male gender seem to play a significant role in increasing early mortality rates [13, 20, 41, 42]. For patients with hip fractures that are treated non-operatively, the reported 30-day mortality rate is significantly higher (34 %) than the 30-day mortality rate of the patients treated operatively [43].

Recent changes in the UK Medical Care in this elderly group of patients led to the improvement of the outcomes of patients with hip fractures. These changes include the national introduction of NHFD, the British Orthopaedic Associations guidelines for fragility fractures and the best practise tariff for hip fractures [5, 6]. The latter includes a geriatrician review for the patient within the first 72 h, operative treatment of the hip fracture within the first 36 h, multispecialty protocol approved by geriatricians, orthopaedics and anaesthetists, evaluation of underlying bone fragility and advanced rehabilitation programmes.

NHFD represents a firmly organised and clinically led quality improvement initiative with main purpose to document different aspects in patients with hip fractures and implement well-established techniques and protocols for the improvement of the outcomes. In the UK, there are 182 hospitals that subscribe in the NHFD and provide monthly data, making the NHFD the largest hip fracture database in the world [6]. Hospital facilities and staff are in continuous evaluation for the implementation of the protocols provided, and audits are performed regularly for multidirectional evaluation of the standard of care provided.

For a better patient assessment and mortality predictor the Nottingham Hip Fracture Score seems to provide important help in regard to 30-day mortality. Variables such as increased age, the presence of 2 or more comorbidities, male gender, low values of haemoglobin and a mental test <6 might be independent predictors of mortality [44]. In another study by Lau et al. [41], it has been reported that the total Charlson comorbidity score (TCCS) correlates with early and late mortality. TCCS is important demonstrating the general condition of the patient on the day of admission after sustaining a hip fracture. Therefore, in patients with multiple comorbidities and high TCCS increased short-term mortality rate is expected. The TCCS is proposed as a reliable predictor of 30-day and 1-year mortality in geriatric patients sustaining a hip fracture [41].

In conclusion, it appears that following the implementation of fast-track care pathways, input from orthogeriatricians, quick patient medical optimisation, combined input by orthopaedic surgeons, anaesthetists, orthogeriatrician and early surgery (earlier than 36 h from the injury) and advanced rehabilitation protocols, there has been a reduction in the 30-day mortality rate of elderly patients undergoing surgery for hip fractures. A number of parameters have contributed to this downward trend noted. Whether we have reached a plateau or whether a steady ongoing reduction in mortality rates will continue is yet to be seen.
